# Gender Difference and Spatial Heterogeneity in Local Obesity

**DOI:** 10.3390/ijerph15020311

**Published:** 2018-02-10

**Authors:** Hee-Jung Jun, Mi Namgung

**Affiliations:** 1 Department of Public Administration and Graduate School of Governance, Sungkyunkwan University, Seoul 03063, Korea; hjun@skku.edu; 2 Department of Urban Engineering, Pusan National University, Busan 46241, Korea

**Keywords:** obesity, spatial heterogeneity, gender difference, spatial analysis, Korea

## Abstract

This study asks if there is gender-specific spatial heterogeneity in local obesity. By using the 2015 Korea Community Health Survey and employing spatial analyses, this study found that there is considerable gender-specific spatial heterogeneity in local obesity rates. More specifically, we found that: (1) local obesity rates are more spatially dependent for women than for men; (2) environmental factors, in general, have stronger effects on local obesity rates for women than for men; (3) environmental factors have more spatially varying effects on local obesity rates for women than for men. Based on these findings, we suggest that policies for obesity prevention should not be based on the assumption of spatial homogeneity and gender indifference, but rather should be refined based on gender-specific spatial heterogeneity in local obesity.

## 1. Introduction

Obesity is currently regarded as a global pandemic and its prevalence has been increasing in both developed and developing countries. Korea is not an exception to this trend as people have engaged in less physical activity and have consumed more energy-dense foods in recent decades [1]. As obesity is associated with an increase in serious health problems such as high blood pressure, diabetes, and heart disease [2,3,4,5,6], it has been the primary cause for increased medical expenditures and a heavier burden for the public health systems [7].

Then, what leads to obesity? Swinburn et al. [8] theorize an obesogenic environment that is defined as “the sum of influences that the surroundings, opportunities, or conditions of life have on promoting obesity in individuals or populations” (p. 564). While there are varying definitions by different scholars, an obesogenic environment is, in general, comprised of physical and socio-economic environment. Physical environment characteristics include access to healthy foods, parks, recreational facilities, and doctors, mixed land use, and denser environment which directly and indirectly affect public health and increase physical activity and walkability [8,9,10,11]. Regarding socio-economic environment, affluent people have more resources to consume healthier foods and engage in further physical activity and educated people are more likely to incorporate healthy behaviors and habits in their lifestyle [8,10,11,12]. Chang et al. [13] also finds that underprivileged areas are more likely to be disordered, for example, with high crime rates, which may discourage physical activity such as walking, thereby leading to obesity.

Among the various studies on obesity, a group of studies pays attention to spatial heterogeneity on obesity. According to Tobler’s [14] First Law of Geography, “Everything is related to everything else, but near things are more related than distant things”. In the context of obesity, Tobler’s statement suggests that obesity can be contagious among individuals and localities through externalities. In fact, studies done in North America suggest that spatial clusters in obesity prevalence are found in the Southern region of the United States and the Saskatchewan region of Canada, and posit that the spatial clusters in the regions are associated with high concentration of blacks and aboriginals [11,15,16,17]. Another group of studies worth noting is about gender difference in obesity. Studies find that, for example, there are higher obesity rates for women than men in the United States [18]. However, in Korea, obesity rates have increased for men in recent years while they remained stable for women, thereby creating a disparity in obesity rates between the men and women [19]. Moreover, studies indicate that individuals’ socio-economic status (e.g., income level, educational attainment, and professional job) is positively related to obesity for men while it is negatively related to obesity for women [1,12,19,20]. Additionally, numerous studies examine the association between race and obesity. For example, studies find racial segregation leads to higher obesity rates as people living in racially segregated communities have limited access to recreational amenities and public transportation and psychological stress (e.g., [13,17]). However, as the level of racial diversity is low in Korea like many other Asian countries compared to Western countries, there have been few studies about the association between race and obesity for Asian countries.

A relatively under-examined area is gender-specific spatial heterogeneity in local obesity. There are three specific reasons why we should look at gender difference and spatial heterogeneity together on local obesity. First, women are more affected by others compared to men. The social contagion model suggests that people’s behaviors are affected by the norms or values of nearby people [21,22]. Thus, in the context of obesity, cultural norms (e.g., overeating food and engaging in less physical activity) that increase obesity can spread obesity across individuals and regions [23]. According to Graziano et al. [24], women are more inclined to be affected by others’ opinions and behaviors compared to men, which suggests that obesity for women is more likely to be spatially contagious. Indeed, studies show that there are more spatial clusters on local obesity for women than for men [10,25].

Second, environmental factors, generally, have stronger effects on obesity for women than for men [26,27,28,29]. In this regard, studies find that less walkable environment and limited access to healthy foods are more likely to be positively associated with obesity for women than for men [26,27,30]. In addition to these physical environment features, studies also suggest a stronger relationship between socio-economic environment and obesity for women than for men [31,32]. The differential associations between women and men may be by reasons of disorders in isolated and deprived environment causing greater psychological stress for women and the increased likelihood of women to engage in overeating from stress compared to men [13,33]. Furthermore, women are more likely to feel anxious and perceive disorder outside, and thus, are less likely to engage in any outdoor activity [34]. Another difference between women and men is the length of their stay in a locality. In nearly all OECD countries, women are less likely to work for a living or participate in any economic activities compared to men [35]. This suggests that women stay in their localities for a longer period, and thus, are more likely to be affected by the local environment.

Third, environmental factors have more spatially varying effects on obesity for women than men. According to Graziano et al. [24], women are more mindful of their appearance and responsive to the behaviors of their friends and neighbors. The responsiveness of women suggests that women are more likely to adjust their weight standards according to community norms. Therefore, the effects of environmental factors depend on the locality where women reside, which in turn leads to more spatial variability of the effect of environmental factors on local obesity for women. There are studies which examine spatial variability of environmental factors on local obesity (e.g., [36,37,38,39]), however, most studies do not examine spatial variability in relation to gender difference. As one of the few studies examining gender difference in spatial variability on local obesity, Christman et al. [10] demonstrate that poverty rate has a positive effect on local obesity in northern New Jersey while it has a negative effect on local obesity in southern New Jersey for women. The same variable has a positive effect on local obesity in northwestern New Jersey but has both positive and negative effects on local obesity in western New Jersey for men.

As mentioned above, most studies do not take into account gender difference and spatial heterogeneity together in analyzing local obesity. However, connecting spatial heterogeneity with gender difference in local obesity can broaden the scope of obesity studies. Thus, we ask the following research question: *Is there gender-specific spatial heterogeneity in local obesity?* As we made theoretical connections between spatial heterogeneity and gender difference in local obesity above, we propose the following hypotheses:

**Hypothesis** **1.** Local obesity is more spatially dependent for women than for men.

**Hypothesis** **2.** Environmental factors have stronger effects on local obesity for women than for men.

**Hypothesis** **3.** Environmental factors have more spatially varying effects on local obesity for women than for men.

## 2. Materials and Methods

### 2.1. Materials

This study aims to analyze gender-specific spatial heterogeneity in local obesity. To test the three hypotheses proposed in the previous section, we used the 2015 Korea Community Health Survey (KCHS). This survey was collected by the Korean Centers for Disease Control and Prevention with cooperation from 253 community health centers which covers the entire country. The survey was collected from people aged 19 years and older. The sample size is 228,558, including 125,729 responses from women and 102,829 responses from men. The KCHS data include a variety of information, including self-reported weight and height that are necessary for measuring obesity and other health parameters (e.g., physical activity, smoking, and alcohol consumption). To be a representative sample, a multi-stage sampling design was employed: first, sub-units within the area that each community health center covers were randomly selected; second, each sub-unit was divided into high-rise apartment complexes and general residential areas; and, finally, households were randomly selected from both high-rise apartment complexes and general residential area. Households were sampled from the official registry of residents.

To measure local obesity rates for women and men, the dependent variable, we first calculated Body Mass Index (BMI)—a classic measure of overweight and obesity—that is a value calculated by dividing the body weight (kilograms) by the square of the body height (meters squared). The World Health Organization (WHO) defines overweight as BMI of 25 kg/m^2^ or higher and obesity as BMI of 30 kg/m^2^ or higher [40]. However, because BMI is generally lower in Asian countries (e.g., [19,36]), we categorized Asian people with BMI of 25 kg/m^2^ or higher as obese. After calculating BMI for all respondents in separate samples of women and men, we calculated local obesity rates—the shares of obese people (BMI of 25 kg/m^2^ or higher)—to the number of respondents in each locality for women and men. In Korea, administrative units are broadly categorized into upper-level and lower-level municipalities. Lower-level municipalities are more intimately associated with local environment that can influence obesity rates. Therefore, we calculated local obesity rates and collected data for local environment at the lower-level municipality. We used local obesity rates only for localities that are within the mainland and omitted those localities that are on islands. Since localities on an island often do not have a neighboring region, it can lower model fitness when running a spatial analysis. Thus, out of 228 localities in the sample, we used 218 localities for the empirical analysis. Table A1 shows the list of 218 localities and local obesity rates of the localities for both women and men.

To examine how environmental factors are differently associated with local obesity rates between women and men, we included physical and socio-economic environmental factors by reviewing previous studies. First, physical environmental factors include population density, level of land-use mix. We used the method of Bhat and Guo [41] to compute the land use mix diversity as follows:

(1) Li=1−{|rL−13|+|mL−13|+|oL−13|43}

where *L_i_* is the mixed land use index, *L* is the total land size, *r* is the size in residential land use, *m* is the size in commercial/industrial land use, and *o* is the size in other land-uses. According to this equation, 1 indicates perfect heterogeneity while 0 indicates perfect homogeneity in land use, area of parks per person, the number of doctors per 1000 people, the number of sports facilities per 1000 people, and the number of fast food restaurants per 1000 people. We included population density, level of land-use mix, and area of parks per person as these factors can potentially enhance walkability, thereby reducing local obesity rates. The numbers of doctors and sports facilities per 1000 people were included as they are directly related to personal health. The number of fast food restaurants per 1000 people was included to capture an environment that can directly increase local obesity. Second, socio-economic environment factors include fiscal self-reliance ratio, percentage of college graduates (among those 25 years or older), percentage of basic living recipients, and percentage of elderly (those 65 years or older). We included fiscal self-reliance ratio to control for local capacity that can influence public health policies and programs. Percentage of college graduates was included as educated people are more likely to adopt a healthy life style and pay attention to their health and well-being. Percentage of basic living recipients was included as a proxy of poverty rate that usually increases obesity rates in developed countries. Finally, percentage of elderly was included to control for demographic structure in each locality.

We collected the data for our study mainly from the Korean Statistical Information Service (KOSIS) and used the data from 2015. The data regarding fast-food restaurants and local fiscal self-reliance ratio were drawn from the Small Business Corporation and Local Finance Integrated Open System, respectively. We used objectively measured data for physical and socio-economic environment to minimize the risk of bias incurred by self-reported survey data. Table A2 presents the summary statistics of the variables for the empirical analysis.

### 2.2. Methods

To test the first hypothesis that local obesity is more spatially dependent for women than for men, we compare the magnitudes of Moran’s I statistic that measures spatial autocorrelation in local obesity rates between women and men. More specifically, Moran’s I statistic calculates the degree of linear association between the values of a variable and the spatially weighted values of the variable at neighboring locations. Spatial modeling needs a spatial weight matrix which defines the spatial structure of analysis units [42]. To create a spatial weight matrix, we looked at several different types of spatial structures, including a contiguity through a common boundary (refers to the rook criterion), vertices (refers to the queen criterion), and 4–6 nearest neighbors. However, Moran’s I statistic is a global test that does not show where clusters are located [43]. Therefore, we additionally employed local indicators of spatial autocorrelation (LISA) to identify specific locations of spatial clusters.

Our second and third hypotheses suggest that environmental factors have stronger effects on local obesity rates for women than for men and have more spatially varying effects on local obesity rates for women than for men. To test these hypotheses, we ran a Geographically Weighted Regression (GWR). A GWR follows the equation:

(2) $$y_{i}=\beta_{0}\left(\upsilon_{i},\nu_{i}\right)+\underset{k}{\sum }\beta_{k}(\upsilon_{i},\nu_{i})x_{ik}+e_{i}$$

where $y_{i}$ is the dependent variable for observation *i*, $x_{ik}$ is the value of an independent variable *k* for observation *i*, (*v_i_, v_i_*), is the location of observation *i*, and $e_{i}$ is the error at point *i*. GWR is a statistical methodology specifically designed for analyzing spatial non-stationarity that the nature and the significance of relationships between variables differ from location to location [44]. In such a way, GWR allows assessing spatial variability of the importance of environmental factors across localities on local obesity rates. In GWR, a weighting scheme that is based on each individual location’s spatial proximity to a location *i* is applied to specific locations (localities in our study) to assign weights. In other words, near locations have more impact on the calibration of coefficients than distant locations, and thus, GWR captures local influences of environmental factors on obesity when spatial heterogeneity is present.

## 3. Results

In this section, we report the empirical results on the proposed hypotheses. We first discuss spatial dependence on local obesity rates, which relates to the first hypothesis. Then, we discuss the associations between environmental factors and local obesity rates, which relates to the second hypothesis. Finally, we discuss spatially varying effects of environmental factors on local obesity rates, which relates to the third hypothesis.

### 3.1. Spatial Dependence on Local Obesity Rates

Spatial dependence on local obesity rates was determined by Moran’s I statistic. A positive Moran’s I value indicates that localities with similar obesity rates are spatially clustered, which signifies uneven distribution of local obesity rates across localities [43]. Moran scatter plots visually illustrate the distribution of local obesity rates for each locality (*x*-axis) in relation to the average local obesity rates of neighboring localities weighted by the spatial weight matrix (*y*-axis). Thus, there can be a fourfold classification: high–high (i.e., localities with higher obesity rates surrounded by other localities with higher obesity rates), low–low (i.e., localities with lower obesity rates surrounded by other localities with lower obesity rates), high–low (i.e., localities with higher obesity rates surrounded by localities with lower obesity rates), and low–high (i.e., localities with lower obesity rates surrounded by localities with higher obesity rates). While a large share of high–high and low–low localities indicate spatial dependence, which increases Moran’s I value, high–low and low–high localities are “spatial outliers”, which lowers Moran’s I value.

**Figure 1:**
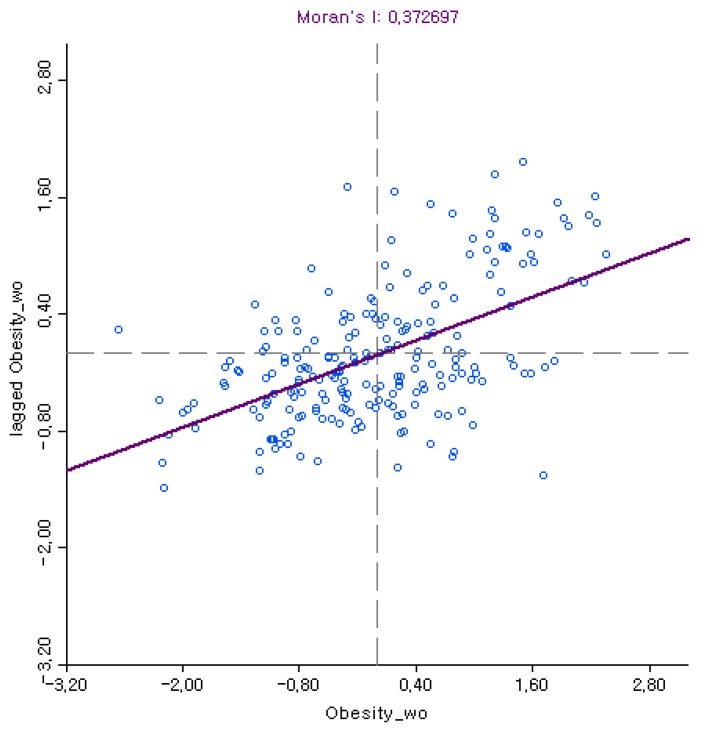
Moran scatterplot: Local obesity rates for women.

**Figure 2:**
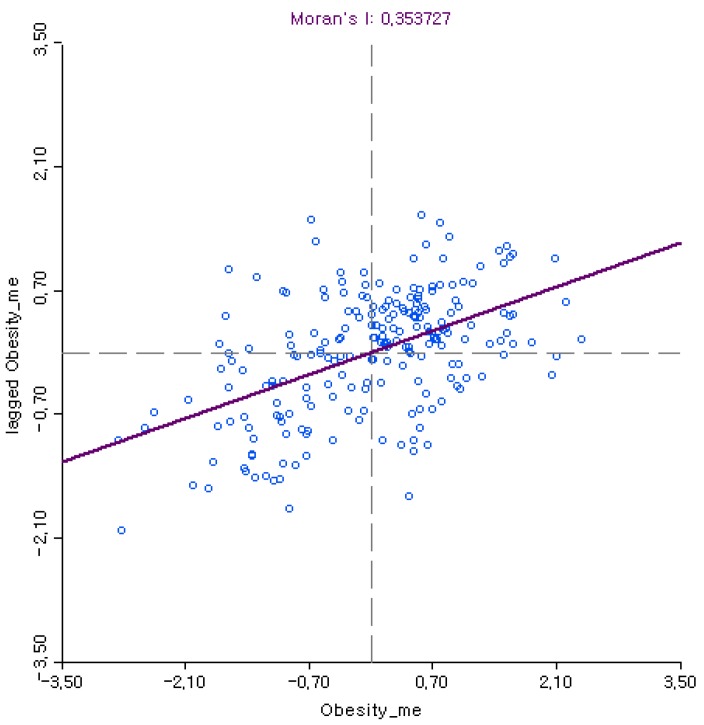
Moran scatterplot: Local obesity rates for men.

As shown in Figure 1 and Figure 2, more localities are placed in high–high and low–low quadrants for both women and men, which generates positive Moran’s I values. More importantly for this study, the higher Moran’s I value for women (0.37) than for men (0.35) based on the rook criterion suggests that local obesity rates are more spatially dependent for women than for men. Moran’s I values of local obesity rates based on other spatial weight matrices also report consistently higher Moran’s I values for women than for men. Table 1 compares Moran’s I values between local obesity for women and men when we used different types of spatial weight matrices.

Furthermore, Moran’s I value of average local BMI based on the rook criterion is higher for women (0.17) than for men (0.10), which corresponds to the higher Moran’s I values of local obesity rates for women than for men. In other words, the greater spatial dependence on local obesity rates for women support the proposed hypothesis that local obesity is more spatially dependent for women than men, which is also consistent with findings in previous studies [10,25].

**Figure 3:**
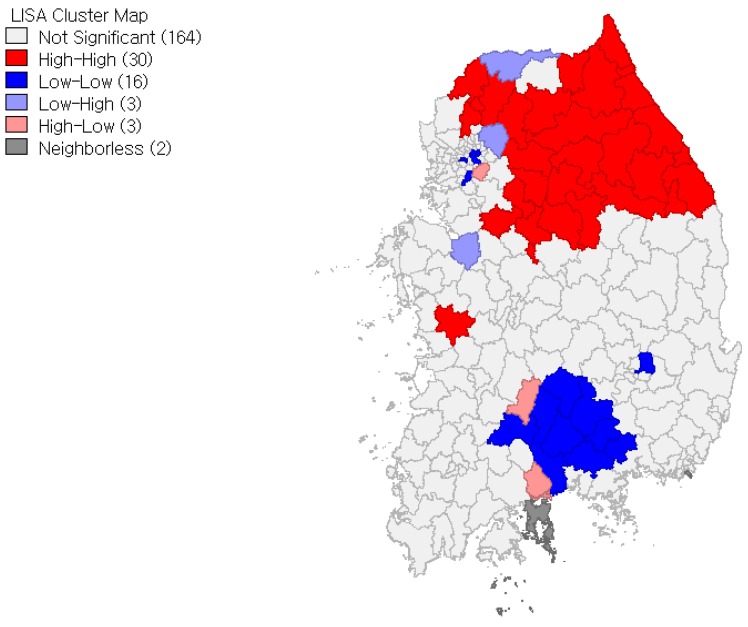
Local Indicators of Spatial Autocorrelation (LISA) Cluster map: Local obesity rates for women.

**Figure 4:**
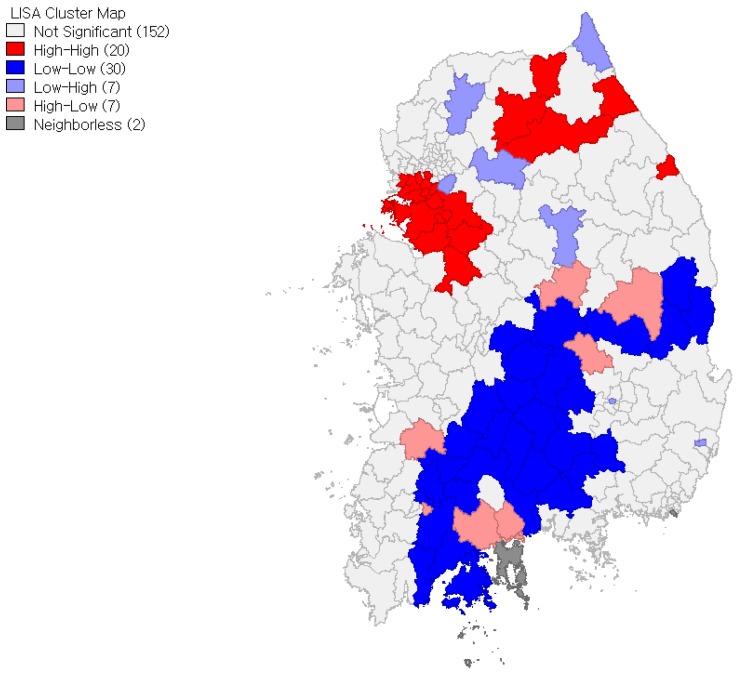
LISA cluster map: Local obesity rates for men.

We also ran LISA that helps us identify specific locations of spatial clusters. In accordance with the positive Moran’s I values, Figure 3 and Figure 4 present more spatial clusters of high-high and low-low localities than high-low and low-high localities for both women and men. (They are significant at *p* = 0.05 significance level. Inference is based on the permutation approach with 999 permutations.) At the same time, the LISA shows geographic differences in local clustering pattern between local obesity rates for women and men. As shown in the figures, high-high localities are clustered heavily in the northeastern region for women, while high–high localities are clustered in both the northeastern and western regions for men. The figures also show that low-low localities are clustered in the central part of the south region for women, while low-low localities are clustered widely throughout the south region for men. All things considered, not only are local obesity rates more spatially dependent for women than for men, but geographic differences in the location of spatial clusters between local obesity rates for women and men also exist.

### 3.2. The Associations between Community Characteristics and Local Obesity Rates

To test the second hypothesis that environmental factors have stronger effects on local obesity rates for women than for men, we employed GWR that can estimate spatial variability in the association between environmental factors and local obesity rates. A GWR estimates locally varying coefficients of independent variables as well as calculates mean values of the locally varying coefficients. Absolute mean values of locally varying coefficients show overall influence of environmental factors on local obesity rates. As shown in Table 2, the higher mean intercept of local obesity rates for men suggest that local obesity rates are, overall, greater for men than for women. However, the absolute mean values of six environmental factors (population density, area of parks per person, number of doctors per 1000 people, number of fast food restaurants per 1000 people, percentage of college graduates, and percentage of basic living recipients) were greater for women than for men. Although the absolute mean values of level of land-use mix, number of sports facilities per 1000 people, financial self-reliance, and percentage of elderly are greater for men than for women, the greater absolute mean values of the other six environmental factors support the second hypothesis that environmental factors have stronger effects on local obesity rates for women than for men.

Table 2 compares the number of localities where each of the environmental factors is statistically significant at a level of 90%. For the variables of population density, area of parks per person, the number of doctors per 1000 people, the number of sports facilities per 1000 people, the number of fast food restaurants per 1000 people, percentage of college graduates, and percentage of basic living recipients, there were more localities where environmental factors are statistically significant on local obesity rates for women than for men. Although not applicable to all of the variables, these results suggest that environmental factors, overall, have stronger effects on local obesity for women than for men as hypothesized. 

Regarding the overall associations between environmental factors and local obesity rates, population density, area of parks per person, percentage of college graduates, and percentage of elderly had negative mean values for both women and men as we expected. Level of land-use mix, the number of sports facilities per 1000 people, fiscal self-reliance ratio, and percentage of basic living recipients had positive mean values for both women and men. While we expected level of land-use mix and the number of sports facilities per 1000 people to have negative associations with local obesity rate, instead, they turned out to have positive associations with local obesity rates, and the variables were not statistically significant in any of the localities. Fiscal self-reliance ratio that was included to capture local capacity for enhancing public health was expected to have a negative association with local obesity, but had positive mean values on local obesity rates for both women and men, thereby requiring further investigation. Percentage of basic living recipients corresponds to the previous study’s finding which showed a positive relationship between poverty rate and obesity. The number of doctors per 1000 people had a negative mean value for women as expected. While the same variable had a positive mean value for men, it was not statistically significant in any of the localities. Similar to the number of doctors per 1000 people, the number of fast food restaurants per 1000 people had a positive mean value for women as expected, but it had a negative mean value for men, and it was not statistically significant in any of the localities.

### 3.3. Spatially Varying Effects of Community Characteristics on Local Obesity Rates

In testing the third hypothesis that environmental factors have more spatially varying effects on local obesity rates for women than for men, we first compared the change in model fitness. As shown in Table 3, the adjusted *R*^2^ in the GWR estimating local coefficients for a variable are significantly higher than those in the global regression—standard OLS regression estimating a global coefficient for a variable. More specifically, for women, the adjusted *R*^2^ is 0.40 in the GWR while it is 0.09 in the global model. For men, the adjusted *R*^2^ is 0.44 in the GWR while it is 0.38 in the global model. The larger growth in the adjusted *R*^2^ for women (0.31 = 0.40 − 0.09) than for men (0.06 = 0.44 − 0.38) indicates that environmental factors have more spatially varying effects on local obesity for women than for men. In addition, there was a larger reduction in AIC—another standard of model fitness—for women (65.41 = 1164.64 − 1099.23) than for men (9.43 = 1105.07 − 1095.64). Not only do these results confirm that the GWR provides a better fit to the data compared to global regression, but they also imply that there are stronger local relationships between environmental factors and local obesity rates for women than for men.

**Figure 5:**
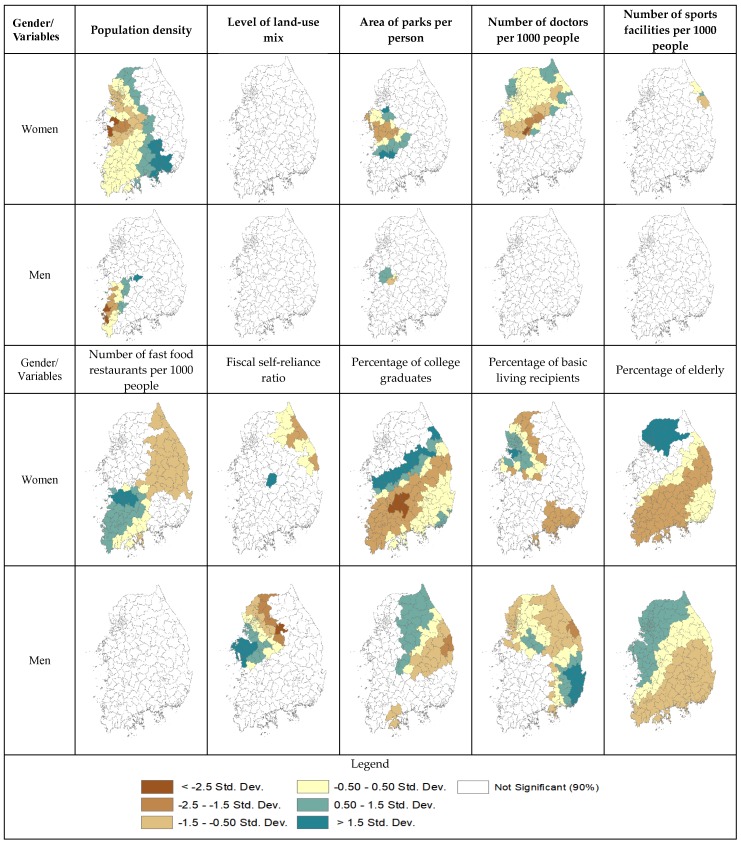
Localities with statistically significant local coefficients in GWR.

Table 3 reports additional GWR results, including means and standard deviations of local coefficients, and minimum and maximum coefficients and their ranges for both women and men. According to “Diff of Criterion” that determines whether a variable is a global (locally fixed) or a local (locally varying) variable, all explanatory variables in GWR were local variables. All of the variables had higher standard deviations of local coefficients for women, which is consistent with larger ranges between minimum and maximum values of local coefficients for women than for men. For instance, the standard deviation of population density is higher for women (0.092) than for men (0.018). In other words, the relationships between environmental factors and local obesity rates are more variable for women than for men across localities. In accordance with the larger standard deviations of local coefficients for women, Figure 5 also shows, environmental factors, in general, have higher levels of regional variation for women than for men. For example, local coefficients of the area of parks per person variable are statistically significant in the west with standard deviations ranging −2.5 to −1.5 and over 1.5 for women while they are statistically significant in the smaller area of the west with standard deviations ranging −1.5 to −0.5 and 0.5 to 1.5. All things considered, the greater increase in model fitness and the larger standard deviations of local coefficients for women than for men support the third hypothesis that environmental factors have more spatially varying effects on local obesity rates for women than for men.

## 4. Discussion

In many countries, obesity has been considered one of the biggest threats to public health, and thus, there have been numerous studies about obesity. Some studies pay attention to gender differences in local obesity while other studies pay attention to spatial heterogeneity in local obesity. Taking into account both gender differences and spatial characteristics in local obesity allows a more strategic institution of public health policies and a more efficient allocation of resources to fix the obesity crisis [11,45]. However, there are only a few studies examining how spatial heterogeneity is differentiated by gender difference. The lack of information in this respect calls for further exploration of the interrelationship between gender difference and spatial heterogeneity in obesity. In this study, we asked if there is gender-specific spatial heterogeneity in local obesity.

Using nationally sampled public health data in Korea and running spatial analyses, we found that there is considerable gender-specific spatial heterogeneity in local obesity. There are three major findings in this study: (1) local obesity rates are more spatially dependent for women than for men; (2) environmental factors, in general, have stronger effects on local obesity rates for women than for men; and (3) environmental factors have more spatially varying effects on local obesity rates for women than for men.

The first finding allows us to reaffirm that spatial dependence on local obesity are important in explaining the difference between women and men. The spatial clusters in local obesity rate that we found suggest that an increase in a locality’s obesity rate can trigger an increase in other surrounding localities’ obesity rates. We found higher Moran’s I values for local obesity rates for women, which reveals a contagious effect across localities far more prominently for women than for men. On the other hand, the spatial clusters in local obesity rate imply that policies to reduce obesity rates in one locality can affect nearby localities, and thus, nearby localities can benefit from the host locality’s policy efforts. Therefore, coordinating local efforts to reduce local obesity rates with neighboring localities can lead to more successful results. In addition, examining spatial clusters can provide us with a focal point or hot spot for starting public health programs to alleviate local obesity. Again, our finding suggests that these spatially dependent relationships in public health policy are more likely to be pronounced for women than for men.

The second finding was supported by our discovery that more environmental factors (i.e., population density, area of parks per person, number of doctors per 1000 people, number of fast food restaurants per 1000 people, percentage of college graduates, and percentage of basic living recipients) have higher absolute mean values of local coefficients on local obesity rates for women than for men. Additionally, we found that there are more localities with environmental factors that are statistically significant on local obesity rates in GWR for women than for men. These results support previous studies’ findings that there is a stronger relationship between community characteristics and obesity for women than for men (e.g., [10,26,27,28,29]). Our study contributes beyond the existing literature as we examine the effect of environmental factors on local obesity rates by running spatial analyses and linking them with gender difference. Our study is also differentiated from previous studies by our examination of the association between environmental factors and local obesity for an Asian country, Korea, rather than a Western country.

The third finding was supported by the increase in the adjusted *R*^2^ from the global regression that estimates a single coefficient for a variable to the GWR that estimates locally varying coefficients for a variable on local obesity rates, but a larger increase for women than men. Additionally, the third finding was supported by wider ranges between minimum and maximum coefficients and larger stand deviations of local coefficients on local obesity rates for women than for men. While the first finding confirms the overall importance of spatial characteristics in analyzing obesity and gender difference and the second finding confirms differential effects of environmental factors on local obesity between women and men, the third finding establishes the notion that spatial variability of environmental factors and gender difference should be taken into account together when analyzing obesity. Therefore, policies for obesity prevention should not be based on the assumption of spatial homogeneity and gender indifference, but rather on gender-specific spatial heterogeneity in local obesity. Not only does this approach enable geographically focused strategies for obesity prevention, but it also allows allocating resources and services based on specific demands based on spatial characteristics and population group.

Our study consists of limitations as in many other obesity studies. We calculated local obesity rates based on self-reported weight and height. People often report lowered weights and increased heights, and thus, there could be an inaccuracy in the BMI that we calculated. Additionally, we could not include objective measures of walkability at the local level as our study covered the entire country and such data were not available. We included population density as a proxy of walkability because sidewalks and public transportation services are more likely to be available in high density areas. However, we cannot rule out the possibility that walkability may be low in high density areas despite the presence of mentioned transportation infrastructure. Using measured weights and heights to calculate BMI and producing more objective measures of walkability at the local level will improve the robustness of the findings in this study. Finally, the results in this study may not be directly applied to other countries since we used data from Korea. Nevertheless, this study provides insights on gender difference and spatial heterogeneity in local obesity to other countries.

Prospective studies may explore “why” there is gender-specific spatial heterogeneity in local obesity. In this study, we focused on “whether” there is gender-specific spatial heterogeneity in local obesity rates by comparing the magnitudes of spatial dependence, the effects of environmental factors, and spatially varying effects of environmental factors between local obesity rates for women and men.

## 5. Conclusions

In the beginning of this study, we asked if there is gender-specific spatial heterogeneity in local obesity rates. We examined this research question further by analyzing a nationally sampled data set from the 2015 Korea Community Health Survey and by employing spatial analyses. The empirical analysis shows that there is a considerable difference in spatial heterogeneity between local obesity rates for women and men, and thus, failing to consider gender difference and spatial heterogeneity in analyzing local obesity can result in biased estimates. Given that obesity is widely known to be a preventable cause of various health problems including diabetes and heart disease [2,3,46,47], we must take into account gender-specific spatial heterogeneity to deal with local obesity more strategically and effectively. Finally, developing countries may be able to initiate a more effective public health policy by referring to the findings in this study because other developing countries, especially, in Asia are expected to take similar paths that people are engaged in less physical activity and consume more energy-dense foods while moving toward developed countries like Korea. Given studies on local obesity have paid less attention to gender-specific spatial heterogeneity, developed countries may also find the results in this study interesting and take into account gender difference and spatial heterogeneity together in studying local obesity.

## Figures and Tables

**Table 1:** Moran’s I Values.

Weight Matrix	Women	Men
Rook criterion	0.37	0.35
Queen criterion	0.36	0.35
4-nearest neighbors	0.35	0.31
5-nearest neighbors	0.35	0.29
6-nearest neighbors	0.34	0.28

**Table 2:** Mean values of local coefficients and the number of locality with significant coefficients in GWR.

Variables	Women		Men	
	Mean	Number of Significant Locality	Mean	Number of Significant Locality
Intercept	24.652	218	35.885	218
Population density (log)	−0.070	157	−0.008	28
Level of land-use mix	0.028	0	0.104	0
Area of parks per person	−0.013	34	−0.005	8
Number of doctors per 1000 people	−0.243	107	0.001	0
Number of sports facilities per 1000 people	0.004	3	0.018	0
Number of fast food restaurants per 1000 people	0.159	77	−0.027	0
Fiscal self-reliance ratio	0.025	17	0.042	83
Percentage of college graduates	−0.110	134	−0.044	50
Percentage of basic living recipients	0.459	107	0.267	149
Percentage of elderly	−0.072	157	−0.241	218

**Table 3:** Summary statistics of local coefficients in GWR.

**Women**	**Mean**	**Standard Deviation**	**Min**	**Max**	**Range**
Intercept	24.652	4.884	18.520	35.237	16.717
Population density (log)	−0.070	0.092	−1.227	0.017	1.243
Level of land-use mix	0.028	1.931	−3.345	3.405	6.749
Area of parks per person	−0.013	0.026	−0.071	0.023	0.094
Number of doctors per 1000 people	−0.243	0.261	−1.205	0.056	1.262
Number of sports facilities per 1000 people	0.004	0.024	−0.052	0.155	0.207
Number of fast food restaurants per 1000 people	0.159	0.258	−0.673	1.008	1.682
Fiscal self-reliance ratio	0.025	0.039	−0.098	0.089	0.188
Percentage of college graduates	−0.110	0.087	−0.290	−0.012	0.278
Percentage of basic living recipients	0.459	0.379	−0.089	1.183	1.272
Percentage of elderly	−0.072	0.138	−0.277	0.202	0.479
*N*	218				
AIC	1099.23	Global regression AIC			1164.64
GWR *R*^2^	0.52	Global regression *R*^2^			0.14
GWR Adj-*R*^2^	0.40	Global regression Adj-*R*^2^			0.09
**Men**	**Mean**	**Standard Deviation**	**Min**	**Max**	**Range**
Intercept	35.885	1.500	33.860	40.260	6.400
Population density (log)	−0.008	0.018	−0.053	0.008	0.061
Level of land-use mix	0.104	1.345	−1.488	3.583	5.071
Area of parks per person	−0.005	0.014	−0.034	0.020	0.055
Number of doctors per 1000 people	0.001	0.102	−0.121	0.179	0.300
Number of sports facilities per 1000 people	0.018	0.012	−0.006	0.047	0.053
Number of fast food restaurants per 1000 people	−0.027	0.042	−0.173	0.068	0.241
Fiscal self-reliance ratio	0.042	0.027	−0.043	0.075	0.118
Percentage of college graduates	−0.044	0.016	−0.077	−0.026	0.051
Percentage of basic living recipients	0.267	0.193	−0.214	0.479	0.693
Percentage of elderly	−0.241	0.042	−0.302	−0.183	0.119
*N*	218				
AIC	1095.64	Global regression AIC			1105.07
GWR *R*^2^	0.52	Global regression *R*^2^			0.41
GWR Adj-*R*^2^	0.44	Global regression Adj-*R*^2^			0.38

## Appendix A

**Table A1:** Local obesity rates (BMI ≥ 25 kg/m^2^).

Locality	Women	Men	Locality	Women	Men	Locality	Women	Men
Jongno (Seoul)	19.22	31.13	Sungnam (Gyeonggi)	23.24	29.94	Taean (Chungcheongnam)	26.51	28.13
Joong (Seoul)	18.43	31.53	Uijeongbu (Gyeonggi)	23.35	33.59	Jeonju (Jeollabuk)	19.14	32.86
Yongsan (Seoul)	19.03	31.59	Anyang (Gyeonggi)	17.50	34.56	Gunsan (Jeollabuk)	23.16	26.88
Seongdong (Seoul)	16.70	31.19	Bucheon (Gyeonggi)	19.59	34.30	Iksan (Jeollabuk)	20.73	30.81
Gwangjin (Seoul)	16.51	33.93	Gwangmyung (Gyeonggi)	19.16	31.49	Jeongup (Jeollabuk)	18.16	32.94
Dongdaemun (Seoul)	20.12	31.52	Pyeongtaek (Gyeonggi)	26.90	37.01	Namwon (Jeollabuk)	18.24	27.61
Jungrang (Seoul)	18.53	31.82	Dongduchun (Gyeonggi)	28.83	36.90	Gimje (Jeollabuk)	14.99	28.35
Seongbuk (Seoul)	22.40	29.15	Ansan (Gyeonggi)	22.70	36.79	Wanju (Jeollabuk)	21.85	28.57
Gangbuk (Seoul)	25.29	31.67	Goyang (Gyeonggi)	17.67	31.10	Jinan (Jeollabuk)	21.26	26.17
Dobong (Seoul)	21.15	31.83	Gwacheon (Gyeonggi)	16.37	31.31	Muju (Jeollabuk)	17.78	27.88
Nowon (Seoul)	16.80	31.58	Guri (Gyeonggi)	17.54	30.46	Jangsu (Jeollabuk)	22.38	25.77
Eunpyeong (Seoul)	17.27	31.73	Namyangju (Gyeonggi)	20.29	34.11	Imsil (Jeollabuk)	15.34	21.41
Seodaemun (Seoul)	17.38	31.80	Osan (Gyeonggi)	20.95	33.26	Sunchang (Jeollabuk)	16.33	26.02
Mapo (Seoul)	21.15	31.72	Siheung (Gyeonggi)	23.54	35.44	Gochang (Jeollabuk)	16.25	33.16
Yangchun (Seoul)	19.34	29.10	Gunpo (Gyeonggi)	16.63	33.42	Buan (Jeollabuk)	17.66	25.65
Gangseo (Seoul)	16.56	32.95	Uiwang (Gyeonggi)	16.57	35.77	Mokpo (Jeollanam)	23.86	36.62
Gooro (Seoul)	21.88	30.71	Hanam (Gyeonggi)	22.80	37.02	Yeosu (Jeollanam)	20.04	25.00
Geumcheon (Seoul)	25.39	35.37	Yongin (Gyeonggi)	16.70	37.15	Suncheon (Jeollanam)	18.18	32.75
Yeongdeungpo (Seoul)	17.61	37.18	Paju (Gyeonggi)	21.33	33.00	Naju (Jeollanam)	21.20	30.15
Dongjak (Seoul)	19.59	31.86	Icheon (Gyeonggi)	23.83	27.49	Gwangyang (Jeollanam)	23.17	31.60
Gwanak (Seoul)	21.36	32.18	Ansung (Gyeonggi)	22.81	35.05	Damyang (Jeollanam)	23.53	26.94
Seocho (Seoul)	12.45	32.31	Gimpo (Gyeonggi)	20.50	32.04	Gokseong (Jeollanam)	17.18	24.38
Gangnam (Seoul)	12.52	35.11	Hwasung (Gyeonggi)	22.27	36.89	Gurye (Jeollanam)	14.82	23.32
Songpa (Seoul)	13.27	33.16	Gwangju (Gyeonggi)	23.21	33.11	Goheung (Jeollanam)	16.63	20.46
Gangdong (Seoul)	16.10	32.93	Yangju (Gyeonggi)	20.72	33.17	Boseong (Jeollanam)	17.26	23.53
Joong (Busan)	22.09	28.54	Pocheon (Gyeonggi)	26.16	29.16	Hwasun (Jeollanam)	19.05	21.83
Seo (Busan)	19.61	32.16	Yeoju (Gyeonggi)	26.04	32.79	Jangheung (Jeollanam)	22.10	26.65
Dong (Busan)	25.83	29.87	Yeonchun (Gyeonggi)	25.20	33.41	Gangjin (Jeollanam)	21.29	28.39
Youngdo (Busan)	20.55	32.76	Gapyeong (Gyeonggi)	24.74	33.10	Haenam (Jeollanam)	10.96	27.06
Jin (Busan)	19.53	31.20	Yangpyeong (Gyeonggi)	23.91	30.88	Yeongam (Jeollanam)	19.37	29.47
Dongrae (Busan)	18.86	28.96	Chuncheon (Gangwon)	21.02	38.97	Muan (Jeollanam)	19.46	31.61
Nam (Busan)	17.48	28.67	Wonju (Gangwon)	23.19	40.05	Hampyeong (Jeollanam)	21.94	30.24
Buk (Busan)	19.30	31.22	Gangneung (Gangwon)	25.75	33.08	Yeonggwang (Jeollanam)	22.38	30.85
Haeundae (Busan)	21.46	33.42	Donghae (Gangwon)	28.46	34.42	Jangseong (Jeollanam)	26.57	25.19
Saha (Busan)	16.31	33.74	Taebaek (Gangwon)	25.86	33.01	Pohang (Gyeongsangbuk)	20.55	33.72
Geomjung (Busan)	21.92	24.88	Sokcho (Gangwon)	24.71	34.13	Gyeongju (Gyeongsangbuk)	19.96	32.75
Gangseo (Busan)	21.78	34.83	Samcheok (Gangwon)	24.57	39.01	Gimcheon (Gyeongsangbuk)	19.01	27.58
Yeonje (Busan)	18.27	34.76	Hongcheon (Gangwon)	27.27	33.73	Andong (Gyeongsangbuk)	20.33	32.38
Suyoung (Busan)	21.80	29.84	Hoengseong (Gangwon)	27.41	30.94	Gumi (Gyeongsangbuk)	22.66	32.93
Sasang (Busan)	20.46	34.17	Yeongwol (Gangwon)	26.36	32.07	Yeongju (Gyeongsangbuk)	20.87	35.18
Gijang (Busan)	22.46	33.58	Pyeongchang (Gangwon)	24.74	30.56	Yeongcheon (Gyeongsangbuk)	23.00	26.88
Joong (Daegu)	18.38	33.50	Jeongseon (Gangwon)	28.18	33.09	Sangju (Gyeongsangbuk)	17.55	25.90
Dong (Daegu)	16.13	34.12	Cheorwon (Gangwon)	19.29	26.25	Mungyeong (Gyeongsangbuk)	20.27	33.51
Seo (Daegu)	21.22	29.81	Hwacheon (Gangwon)	27.52	37.15	Gyeongsan (Gyeongsangbuk)	16.35	33.10
Nam (Daegu)	19.73	27.36	Yanggu (Gangwon)	25.77	36.53	Gunwi (Gyeongsangbuk)	18.65	27.08
Buk (Daegu)	18.88	33.03	Inje (Gangwon)	27.00	39.40	Uiseong (Gyeongsangbuk)	14.79	25.07
Susung (Daegu)	13.48	30.00	Goseong (Gangwon)	22.37	28.57	Cheongsong (Gyeongsangbuk)	17.54	25.69
Dalseo (Daegu)	18.65	38.80	Yangyang (Gangwon)	28.39	34.16	Yeongyang (Gyeongsangbuk)	17.88	24.55
Dalsung (Daegu)	21.01	32.59	Chungju (Chungcheongbuk)	25.00	32.45	Yeongdeok (Gyeongsangbuk)	20.04	27.49
Dong (Incheon)	26.04	27.71	Jeocheon (Chungcheongbuk)	24.61	30.81	Cheongdo (Gyeongsangbuk)	23.94	25.88
Nam (Incheon)	20.68	30.15	Cheongju (Chungcheongbuk)	20.92	31.14	Goryeong (Gyeongsangbuk)	20.79	27.36
Yeonsu (Incheon)	19.19	37.95	Boeun (Chungcheongbuk)	19.10	29.30	Seongju (Gyeongsangbuk)	23.49	28.32
Namdong (Incheon)	22.98	32.93	Okcheon (Chungcheongbuk)	17.02	27.25	Chilgok (Gyeongsangbuk)	20.79	30.94
Bupyeong (Incheon)	21.49	32.53	Youngdong (Chungcheongbuk)	19.27	26.04	Yecheon (Gyeongsangbuk)	19.28	24.68
Gyeyang (Incheon)	24.26	31.58	Jincheon (Chungcheongbuk)	24.94	32.20	Bonghw (Gyeongsangbuk)	20.20	27.59
Seo (Incheon)	18.66	32.69	Goesan (Chungcheongbuk)	22.52	24.60	Uljin (Gyeongsangbuk)	21.19	26.60
Dong (Gwangju)	19.16	33.18	Eumseong (Chungcheongbuk)	25.12	36.79	Jinju (Gyeongsangnam)	17.12	27.21
Seo (Gwangju)	13.67	31.77	Danyang (Chungcheongbuk)	27.99	33.42	Tongyeong (Gyeongsangnam)	22.11	34.90
Nam (Gwangju)	18.77	31.25	Jeungpyeong (Chungcheongnam)	24.46	28.93	Sacheon (Gyeongsangnam)	19.84	28.33
Buk (Gwangju)	19.18	30.59	Cheonan (Chungcheongnam)	20.00	34.07	Gimhae (Gyeongsangnam)	21.18	34.68
Gwangsan (Gwangju)	17.83	30.30	Gongju (Chungcheongnam)	23.99	29.72	Miryang (Gyeongsangnam)	18.90	29.80
Dong (Daejeon)	24.03	34.58	Boryong (Chungcheongnam)	25.29	33.25	Yangsan (Gyeongsangnam)	20.16	36.17
Joong (Daejeon)	19.60	33.98	Asan (Chungcheongnam)	18.00	34.53	Changwon (Gyeongsangnam)	17.36	32.47
Seo (Daejeon)	14.84	31.53	Seosan (Chungcheongnam)	15.95	27.79	Uiryeong (Gyeongsangnam)	16.10	27.37
Yusung (Daejeon)	15.29	32.74	Nonsan (Chungcheongnam)	23.27	29.30	Haman (Gyeongsangnam)	17.61	28.35
Daedeok (Daejeon)	20.99	36.74	Gyeryong (Chungcheongnam)	18.11	34.82	Changnyeong (Gyeongsangnam)	15.85	29.46
Joong (Ulsan)	19.45	25.00	Dangjin (Chungcheongnam)	18.74	34.11	Goseong (Gyeongsangnam)	19.10	29.50
Nam (Ulsan)	21.40	33.80	Geumsan (Chungcheongnam)	20.37	25.00	Hadong (Gyeongsangnam)	16.86	26.05
Dong (Ulsan)	21.15	28.76	Buyeo (Chungcheongnam)	21.48	29.82	Sancheong (Gyeongsangnam)	12.62	24.16
Buk (Ulsan)	16.21	35.84	Seocheon (Chungcheongnam)	24.55	34.53	Hamyang (Gyeongsangnam)	12.74	25.67
Ulju (Ulsan)	17.00	34.37	Cheongyang (Chungcheongnam)	22.24	27.93	Geochang (Gyeongsangnam)	17.00	27.23
Sejong (Sejong)	17.45	33.87	Hongsung (Chungcheongnam)	20.36	31.51	Hapcheon (Gyeongsangnam)	13.76	20.32
Suwon (Gyeonggi)	18.07	34.07	Yesan (Chungcheongnam)	23.27	30.23			

**Table A2:** Summary Statistics of the Explanatory Variables.

Variables	*N*	Mean	S.D.	Min	Max
Local obesity rate for women	218	20.415	3.566	10.959	28.834
Local obesity rate for men	218	31.092	3.771	20.317	40.049
Population density (log)	218	4087.330	6287.769	19.855	28,260.740
Level of land-use mix	218	0.337	0.157	0.073	0.763
Area of parks per person	218	21.120	22.225	0.397	172.342
Number of doctors per 1000 people	218	2.536	2.317	0.810	22.010
Number of sports facilities per 1000 people	218	2.080	6.353	0.368	94.951
Number of fast food restaurants per 1000 people	218	0.510	0.271	0.019	2.353
Fiscal self-reliance ratio	218	28.368	12.275	10.230	64.510
Percentage of college graduates	218	33.843	16.469	8.355	179.409
Percentage of basic living recipients	218	3.762	1.806	0.681	9.101
Percentage of elderly	218	23.191	10.738	7.953	50.061
